# Semi-Supervised Traffic Sign Detection with Dynamic Pseudo-Label Selection and Gated Feature Fusion-Based Proposal Refinement

**DOI:** 10.3390/s26123836

**Published:** 2026-06-16

**Authors:** Chenhui Xia, Yeqin Shao, Meiqin Che, Guoqing Yang

**Affiliations:** 1 School of Transportation and Civil Engineering, Nantong University, Nantong 226019, China; 2333320012@stmail.ntu.edu.cn (C.X.); meiqin.che@ntu.edu.cn (M.C.); 2 Suzhou Research Institute of Industrial Technology, Zhejiang University, Hangzhou 310058, China; ygq78@zju.edu.cn

**Keywords:** semi-supervised learning, traffic sign detection, pseudo-label selection, imbalanced class distribution, feature fusion

## Abstract

Accurate traffic sign detection is important for the safety of autonomous driving systems. However, fully supervised methods require a large amount of manual annotation, which is cost-prohibitive and time-consuming. Semi-supervised methods employ a small amount of labeled data and a large amount of unlabeled data to train the models, hence largely reducing the annotation costs. However, these methods have the following challenges: (1) with an imbalanced long-tail class distribution of traffic signs, they tend to achieve poor performance on tail classes; (2) they often fail to detect small traffic signs. To solve these issues, we propose a Semi-Supervised Traffic Sign Detection method with Dynamic Pseudo-Label Selection and Gated Feature Fusion-based Proposal Refinement. Firstly, we design a Class Distribution-based Dynamic Pseudo-Label Selection module (CD-DPLS) to select pseudo-labels for different classes based on the class distribution information, which reduces the tendency to select more pseudo-labels from head classes instead of tail classes, thereby improving the tail class detection performance. Secondly, we employ a Gated Feature Fusion-based Proposal Refinement strategy (GFF-PR) to refine detection proposals by fusing different-scale features with a gating mechanism, which facilitates the detection of small traffic signs. In addition, we use an Adaptive-Weight Focal Loss (AWFL), with which the weight of each pseudo-label is determined by the ratio between its classification confidence and the corresponding class-specific classification-confidence threshold. Experiments on traffic sign datasets demonstrate that the proposed method outperforms state-of-the-art semi-supervised approaches, with mAP_50_ scores of 10.8% and 34.9% using only 1% and 10% labeled data, respectively.

## 1. Introduction

Traffic signs play a crucial role in road scenes by delivering regulatory instructions, warning information, and navigation guidance to drivers. Accurate perception of these signs is fundamental for maintaining driving safety, since missed or incorrect detections may lead to dangerous driving behaviors and traffic accidents. With the rapid development of intelligent transportation technologies, especially autonomous driving systems and Advanced Driver Assistance Systems (ADAS), robust traffic sign detection has attracted increasing attention in recent years [1].

Benefiting from the emergence of large-scale annotated datasets, deep learning-based object detection approaches have achieved remarkable progress across a variety of visual tasks [2,3,4,5,6,7]. Fully supervised detectors generally rely on abundant manually labeled samples to achieve strong detection performance. Nevertheless, acquiring precise annotations is highly time-consuming and expensive, particularly in traffic sign detection scenarios where objects are usually small and require accurate bounding-box labeling.

To alleviate the reliance on large quantities of annotated samples, semi-supervised learning (SSL) has become an active research direction [8,9,10,11]. SSL methods utilize limited labeled data together with a substantial amount of unlabeled data to improve model generalization capability. By leveraging the hidden information contained in unlabeled samples, these approaches can effectively reduce annotation costs while maintaining competitive detection accuracy. Existing semi-supervised approaches are commonly divided into two mainstream paradigms, namely, consistency regularization and pseudo-label-based learning [12,13,14,15,16,17,18,19,20,21,22].

Consistency regularization methods encourage the model to produce stable predictions under different perturbations or augmentations of the same input sample. Although such strategies can improve feature smoothness and enhance generalization performance, their effectiveness strongly depends on the quality of supervisory signals. When pseudo-labels contain incorrect predictions caused by class imbalance or noisy samples, the consistency constraint may further amplify these errors during training.

**Figure 1:**
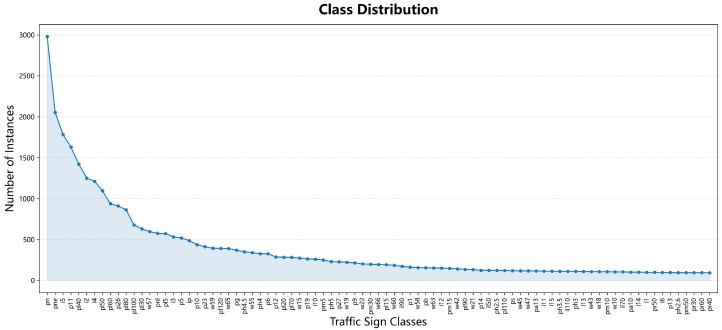
Imbalanced class distribution in the traffic sign dataset. The horizontal axis shows the classes of traffic signs, and the vertical axis shows the number of traffic sign instances, revealing a severe imbalance between head classes and tail classes.

Existing pseudo-labeling methods in traffic scenes usually rely on a single classification-confidence threshold for all object classes. However, this simple strategy ignores the difference in classification confidences of object classes, which is due to the imbalanced class distribution. As shown in Figure 1, the traffic sign dataset exhibits imbalance in instance counts of different classes, where the classes on the left appear much more frequently than those on the right. This is characterized by imbalanced class distribution. Here we take the 18 classes with more than 500 instances as head classes and the remaining 66 classes with fewer than 500 instances as tail classes. Since the model mainly learns from the head classes, it tends to predict these classes with higher classification confidences. In contrast, due to insufficient training, the model tends to predict tail classes with lower classification confidences. Therefore, a fixed threshold will filter out many potential positive samples from tail classes and treat them as background, which will lead to a severe loss of useful supervision information from tail classes.

To address these challenges, we propose a semi-supervised traffic sign detection method with dynamic pseudo-label selection and Gated Feature Fusion-based Proposal Refinement. Built on YOLOv11, the proposed method introduces class-aware pseudo-label selection, gated cross-scale feature fusion, and adaptive loss weighting to reduce the tendency to select more pseudo-labels from head classes instead of tail classes, improving the detection performance of small traffic signs.

It is worth noting that, recent advances in the YOLO family further motivate our design choice. The YOLO series has evolved rapidly in 2024–2025: YOLOv10 [23] introduced NMS-free consistent dual assignments, YOLOv12 [24] developed area attention and R-ELAN, YOLOv13 [25] exploited hypergraph-based high-order correlations, and YOLOv26 [26] focused on NMS-free edge inference. Despite these advances, we select YOLOv11 as our detection backbone due to its strong balance between efficiency and representational power. In particular, its attention-enhanced C3k2 module and C2PSA block provide strong multi-scale feature discrimination, which benefits the detection of distant and small traffic signs. To further ensure fairness and rule out the possibility that performance gains are merely attributed to a stronger backbone, we additionally re-implement the semi-supervised Efficient Teacher framework on YOLOv11 for comparison.

This work extends our previous semi-supervised traffic sign detection framework [27], which introduces the Dual Confidence Fusion Module (DC-Fusion), Structured Block-Regularized Neck network (SBR-Neck), and Spatial-Context-Aware Upsampling (SCA-Upsampling) modules to optimize the baseline Efficient Teacher by refining pseudo-labels and alleviating target detail loss. Building upon the previous framework, this paper further presents three new components specifically designed to tackle the challenges of imbalanced class distributions and small traffic sign detection: the Class Distribution-based Dynamic Pseudo-Label Selection module (CD-DPLS), the Gated Feature Fusion-based Proposal Refinement strategy (GFF-PR), and the Adaptive-Weight Focal Loss (AWFL).

The main contributions of this paper are summarized as follows:

**(1) We propose a Class Distribution-based Dynamic Pseudo-Label Selection module (CD-DPLS).** Instead of using a single global classification-confidence threshold for all classes, the CD-DPLS assigns different classification-confidence thresholds to different traffic sign classes to avoid retaining more pseudo-labels for head classes while filtering out tail classes. To make the class distribution estimation more reliable under limited labeled data, we combine two kinds of class distributions: the class distribution from labeled data and the class distribution provided by CLIP on unlabeled data. Thus, the proposed method can adjust class-specific classification-confidence thresholds dynamically to improve the detection performance on tail classes.

**(2) We propose a Gated Feature Fusion-based Proposal Refinement strategy (GFF-PR).** To avoid the missed detections of small traffic signs, the GFF-PR fuses different-scale features through a gating mechanism, and it uses the fused features to refine object proposals. Specifically, the GFF-PR uses two types of proposals based on the fused feature pyramid for training. The first type of proposals consists of those whose fused confidence (computed as the product of classification confidence and localization confidence) exceeds a dynamic positive threshold determined from the mean and standard deviation of fused confidences in the current batch. The second type of proposals consists of those whose fused confidence lies between the dynamic positive threshold and the fixed negative threshold of 0.1. These proposals are retained only when the confidence gain between the fused feature pyramid and original feature pyramid exceeds 0.2. This strategy thereby improves the detection performance of small traffic signs.

**(3) We propose an Adaptive-Weight Focal Loss (AWFL).** Existing methods usually treat all pseudo-labels above the classification-confidence threshold equally, while the AWFL assigns each pseudo-label an adaptive weight based on the ratio between its classification confidence and the class-specific classification-confidence threshold. As a result, pseudo-labels, which are much higher than the aforementioned threshold, can get larger weights. This mitigates the issues caused by the imbalanced class distributions.

## 2. Related Work

### 2.1. Semi-Supervised Object Detection

Semi-Supervised Object Detection (SSOD) has developed rapidly in recent years by extending semi-supervised learning strategies originally designed for image classification tasks. Early studies mainly relied on self-training frameworks, where pseudo-labels generated from unlabeled samples are iteratively used for detector optimization. Among these methods, STAC [28], inspired by the Noisy Student paradigm, adopts a two-stage training procedure in which a pretrained teacher detector first produces pseudo-labels, followed by supervised training of the student detector using both labeled data and generated annotations.

Although this staged paradigm demonstrates promising performance, the fixed teacher mechanism often suffers from limited pseudo-label quality and error accumulation. To address these issues, subsequent studies introduced teacher–student collaborative optimization frameworks [16,29,30,31,32]. Most of these approaches employ an Exponential Moving Average (EMA) strategy to continuously update the teacher network using the student parameters, thereby producing more reliable pseudo-labels throughout the training process.

Based on this dynamic framework, numerous methods have explored different strategies for improving pseudo-label accuracy and reducing training bias. For example, Unbiased Teacher [29] mitigates category imbalance through bias correction mechanisms, whereas Instant-Teaching [16] enhances the interaction between teacher and student models to suppress error propagation. Soft Teacher [31] improves training stability by incorporating score-aware classification losses and box jittering strategies. More recently, Transformer-based SSOD frameworks have also emerged. Semi-DETR [32] extends semi-supervised learning to DETR architectures by introducing hybrid bipartite matching and cost-guided pseudo-label filtering mechanisms.

Different from the above studies, our method primarily focuses on resolving pseudo-label ambiguity and inaccurate assignment issues in semi-supervised single-stage traffic sign detection frameworks.

### 2.2. Traffic Sign Detection

Early traffic sign detection approaches mainly relied on manually designed pipelines [33]. These methods typically first extracted candidate regions according to low-level visual cues, including color distributions, geometric shapes, and edge responses, and subsequently applied traditional classifiers such as Support Vector Machines (SVM) and AdaBoost for category recognition using handcrafted descriptors like Histogram of Oriented Gradients (HOG) [34,35]. However, the dependence on manually engineered features limits the adaptability of such methods under complex environments involving illumination variation, occlusion, and cluttered backgrounds.

The emergence of deep convolutional neural networks has significantly advanced traffic sign detection by enabling automatic hierarchical feature extraction from raw images [36]. Recent studies have further concentrated on enhancing feature representation capability for small-scale and multi-scale traffic signs. Wang et al. [37] proposed AF-FPN, which strengthens multi-scale feature fusion through adaptive attention mechanisms and feature enhancement operations with varying receptive fields. To better capture long-range contextual information, Omid et al. [38] introduced a pyramid Transformer architecture integrating local attention and multi-head self-attention (MHSA) [39]. In addition, Wang et al. [40] designed a context-aware weighted fusion framework combining convolutional operations, MHSA, and relative positional encoding to improve adaptive global–local feature interaction. Zhang et al. [41] further proposed a cascaded R-CNN-based detector with hierarchical feature refinement and attention-guided multi-scale representation learning, achieving improved robustness against scale variation and complicated road backgrounds.

Despite the remarkable progress achieved by existing traffic sign detectors, most current methods still rely heavily on large-scale annotated datasets, which restricts their applicability in scenarios with limited labeling resources.

## 3. Methods

### 3.1. Overview of Our Method

**Figure 2:**
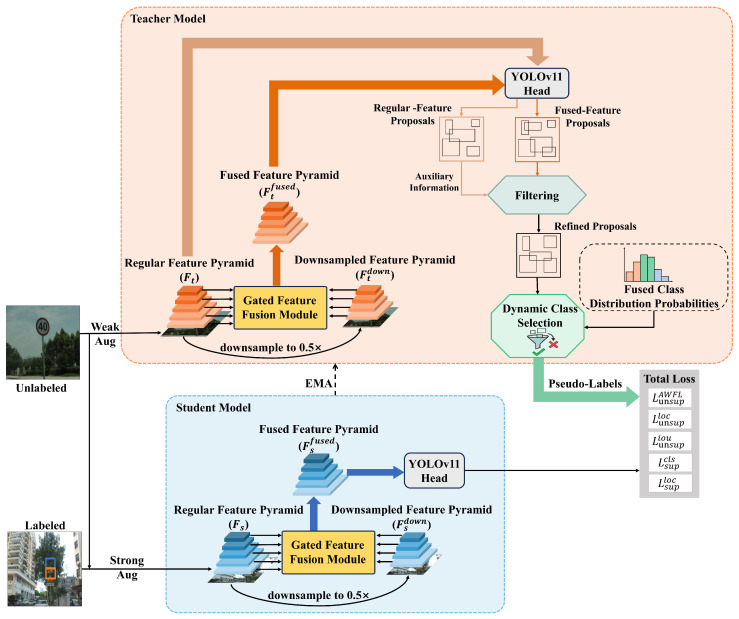
The overall architecture of the proposed semi-supervised traffic sign detection framework. The framework is based on the Efficient Teacher paradigm using YOLOv11 as the baseline. It contains two parallel streams: the teacher model generates pseudo-labels from weakly augmented unlabeled data, while the student model learns from labeled and unlabeled data under stronger augmentation. The framework includes three key components: the Class Distribution-based Dynamic Pseudo-Label Selection module (CD-DPLS), which dynamically sets class-specific pseudo-label classification-confidence thresholds; the Gated Feature Fusion-based Proposal Refinement strategy (GFF-PR), which fuses multi-scale features to refine proposals and recover small traffic signs; and the Adaptive-Weight Focal Loss (AWFL), which mitigates the issues caused by the imbalanced class distributions.

The detailed architecture of the proposed semi-supervised framework is clearly illustrated in Figure 2.

We divide the training data into a small set of labeled data $D_{L}={\left\{\left(x_{i}^{l},y_{i}^{l}\right)\right\}}_{i=1}^{N_{l}}$ and a large set of unlabeled data $D_{U}={\left\{x_{i}^{u}\right\}}_{i=1}^{N_{u}}$, typically satisfying $N_{u}\gg N_{l}$.

The framework consists of a teacher model and a student model with identical structures. The student model updates its parameters via backpropagation, while the teacher model’s parameters are updated using the Exponential Moving Average (EMA) of the student model’s parameters to ensure stability in pseudo-label generation.

### 3.2. Class Distribution-Based Dynamic Pseudo-Label Selection

Existing pseudo-label selection methods mainly rely on fixed confidence estimation to filter unreliable predictions. Although such strategies can improve the quality of pseudo-labels to some extent, they are still insufficient for traffic sign detection under class imbalance. In particular, teacher models tend to produce higher classification confidences for head classes, while the classification confidences of tail classes are lower due to limited training samples. Therefore, using a fixed global threshold or a simple Top-k strategy will suppress the tail classes, introducing pseudo-label noise for head classes. This issue may accumulate during iterative semi-supervised training, further exacerbating the model’s preference for head classes.

**Figure 3:**
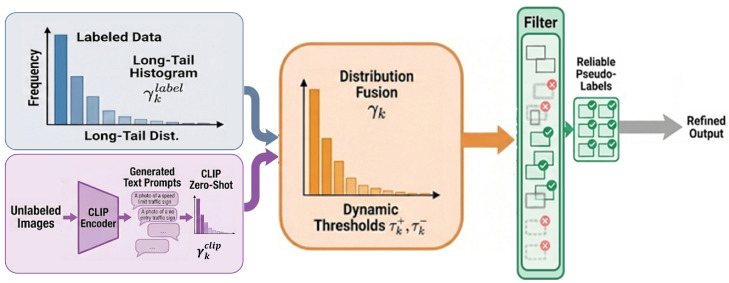
Schematic illustration of the Class Distribution-based Dynamic Pseudo-Label Selection module (CD-DPLS). The CD-DPLS combines the class distribution from labeled data with the class distribution estimated by CLIP from unlabeled data. By using the fused class distribution, the module dynamically adjusts the classification-confidence threshold for each class.

More importantly, using fixed classification-confidence thresholds alone cannot explicitly regulate the distribution of pseudo-labels. As a result, even after low-quality pseudo-labels are filtered out, the generated pseudo-labels may still remain imbalanced across classes, which prevents the detector from learning tail classes. To address this issue, we propose the Class Distribution-based Dynamic Pseudo-Label Selection (CD-DPLS) module, as illustrated in Figure 3. Instead of applying fixed classification-confidence thresholds to all classes, the CD-DPLS exploits the class distribution derived from labeled data and unlabeled data to dynamically adjust the classification-confidence threshold for each class. The CD-DPLS improves the detection performance on tail classes.

#### 3.2.1. Theoretical Basis and Distribution Estimation

The effectiveness of class distribution probabilities is grounded in a statistical observation: in semi-supervised learning settings, even when the proportion of labeled data is small, the empirical class distribution of the labeled data can still approximate the class distribution characteristics of the entire dataset.

Assuming the labeled dataset $D_{L}$ and the unlabeled dataset $D_{U}$ are independent and identically distributed, according to the generalization of Hoeffding’s inequality, for any class *k*, the deviation between the estimated class distribution probability $\gamma_{k}$ and the true distribution $\gamma_{k}^{*}$ satisfies the following probability bound:

(1) $$\left|\gamma_{k}-\gamma_{k}^{*}\right|\leq \sqrt{\frac{\log(2n)}{2n}}+\sqrt{\frac{\log(2m)}{2m}}$$

where *n* and *m* denote the numbers of labeled and unlabeled data, respectively. As the number of labeled samples *n* increases, this error converges at a rate of $O_{p}\left(1/\sqrt{n}\right)$.

However, this distribution estimation approach based on labeled data still has clear limitations in long-tailed semi-supervised scenarios. On the one hand, when the labeling ratio is low, the number of samples from tail classes in the labeled set is often limited. As a result, although the overall error bound decreases as *n* grows, the estimated class probabilities for different classes may still fluctuate. On the other hand, the unlabeled data are usually more abundant than the labeled data and thus contain richer information. Relying only on the labeled set makes it difficult to fully capture the latent distribution characteristics of the entire training dataset. Therefore, we calibrate the dataset’s class distribution by combining the class distribution probabilities derived from labeled data with those from unlabeled data estimated by a pretrained CLIP, ultimately obtaining more reliable class distribution probabilities for the subsequent dynamic selection process.

#### 3.2.2. CLIP-Based Class Distribution Estimation

Since class labels in traffic sign detection tasks are often abbreviations (e.g., “pl50”, “p23”) and lack semantic information, directly applying a pretrained CLIP for zero-shot inference faces challenges. To this end, we designed a “Class-Semantic Mapping Mechanism” to utilize CLIP’s generalization ability to extract class distribution probabilities $\gamma_{k}^{clip}$ from unlabeled data, which serve as a complement to the class distribution probabilities $\gamma_{k}^{label}$ derived from labeled data.

Since the original dataset labels (e.g., “pl50”, “p23”) are engineering codes, the CLIP text encoder cannot directly capture their semantics. Therefore, we construct a mapping function M that maps the abbreviated label space $C_{\mathrm{abbr}}$ to the natural language description space $C_{\mathrm{desc}}$. For example, “pl50” is mapped to “Speed limit 50 km/h”, and “p23” is mapped to “No left turn”. To enhance the contextual representation of textual features, we insert the mapped descriptions into a prompt template to generate the text embedding $t_{k}$ for each class *k*:

(2) $$t_{k}={\;\mathrm{Encoder}\;}_{\mathrm{text}\;}\left(“\;A\;\mathrm{photo}\;\mathrm{of}\;a\;\left[“+M\left({\;\mathrm{class}\;}_{k}\right)+”\right]\;\mathrm{traffic}\;\mathrm{sign}”\;\right)$$

Then we use the CLIP to perform sampling inference on the unlabeled dataset $D_{U}$ to estimate its distribution. Specifically, for unlabeled images, we use the preliminary prediction boxes from the teacher model to crop them and input them into the CLIP image encoder to extract visual features $v_{i}$. By calculating the cosine similarity between the visual features and all class text embeddings $t_{k}$, we obtain the semantic prediction probability for the sample:

(3) $$p_{k}^{clip}\left(v_{i}\right)=\frac{exp\left(sim\left(t_{k},v_{i}\right)/\tau \right)}{\sum_{j=1}^{K}\;exp\left(sim\left(t_{j},v_{i}\right)/\tau \right)}$$

By aggregating statistics on a subset of unlabeled samples, we obtain the distribution estimation $\gamma_{k}^{clip}$ based on semantic understanding.

By counting the predicted class of unlabeled data, we obtain the class distribution probabilities of the unlabeled data, denoted as $\gamma_{k}^{clip}$. Finally, to combine the class distribution probabilities derived from labeled and unlabeled data, we employ a weighted summation to fuse the two probabilities, yielding the final fused class distribution probability $\gamma_{k}$:

(4) $$\gamma_{k}=\beta \;\cdot \;\gamma_{k}^{\mathrm{label}\;}+\left(1-\beta \right)\;\cdot \;\gamma_{k}^{\mathrm{clip}\;}$$

#### 3.2.3. Class Distribution-Based Threshold Setting

To achieve better alignment between the pseudo-label distribution and the true distribution, we formulate the semi-supervised learning process as an optimization problem with regularization constraints. Unlike traditional methods that only minimize classification loss, we introduce a class-aware regularization term to explicitly control the quantity of pseudo-labels generated for each class.

Specifically, for class *k*, the teacher model predicts the classification confidences for the corresponding traffic sign on the unlabeled dataset, and these confidences are sorted in descending order as $Conf_{k,1}\geq Conf_{k,2}\geq \ldots \geq Conf_{k,m}$. Additionally, we further introduce positive- and negative-sample reliability ratios $\eta_{1},\eta_{0}\in \left[0,1\right]$ to filter out the false positive samples. The top $\eta_{1}\gamma_{k}$ proportion of samples are selected as positive pseudo-labels, while the bottom $\eta_{0}\left(1-\gamma_{k}\right)$ proportion are selected as negative pseudo-labels. Thereby, the classification-confidence thresholds of positive and negative samples are computed as follows:

(5) $$\tau_{k}^{+}={Conf}_{k,\left\lfloor \eta_{1}\gamma_{k}m\right\rfloor },\;\tau_{k}^{-}={Conf}_{k,\left\lfloor {m-\eta }_{0}\left(1-\gamma_{k}\right)m\right\rfloor }$$

Under this definition, $\tau_{k}^{+}$ and $\tau_{k}^{-}$ vary dynamically with the class distribution probability to improve the detection accuracy of each class.

#### 3.2.4. Dynamic Pseudo-Label Selection

Finally, the classification pseudo-label ${\hat{y}}_{k}$ for an unlabeled sample *x* by the teacher model is determined by the following rule:

(6) $${\hat{y}}_{k}=\left\{\begin{array}{cc} 1, & {\mathrm{Conf}}_{k}\left(x\right)\geq \tau_{k}^{+}\\ 0, & {\mathrm{Conf}}_{k}\left(x\right)\leq \tau_{k}^{-}\\ \mathrm{ignore}, & \mathrm{otherwise} \end{array}\right.$$

This mechanism encourages the pseudo-label distribution of each class to better align with the target class distribution.

### 3.3. Gated Feature Fusion-Based Proposal Refinement Strategy

In the previous section, we introduced the CD-DPLS to address the challenge of imbalanced class distributions. However, in semi-supervised object detection, pseudo-label quality is also limited by the missed detection of small objects. The small objects often obtain low classification confidences due to their limited visual information, making them more likely to be mistaken as background. As a result, many potentially useful positive samples are ignored during the training.

To address this issue, we propose the Gated Feature Fusion-based Proposal Refinement strategy (GFF-PR). The main idea is to enhance the feature representation of small traffic signs by adaptively fusing features from different pyramid levels. Based on the fused feature pyramid, the model generates proposals for training and further refines them. In this way, proposals, which are weak in the original feature pyramid but become more reliable after feature fusion, can be identified and retained as valuable training signals.

#### 3.3.1. Feature Pyramid Construction for Feature Fusion

**Figure 4:**
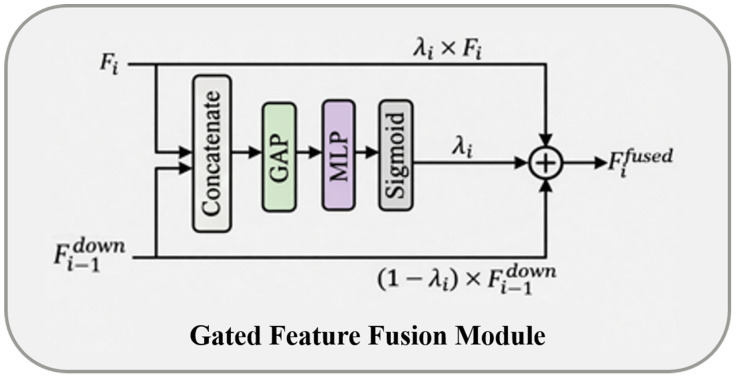
Detailed architecture of the Gated Feature Fusion module. This module builds a fused feature pyramid by adaptively combining features from different scales. Specifically, it takes the original feature map $F_{i}$ and the downsampled feature $F_{i-1}^{down}$ as inputs, and it feeds the concatenated result into a lightweight gating network, composed of Global Average Pooling and a Multi-Layer Perceptron to generate the fusion weight $\lambda_{i}$. The final fused feature $F_{i}^{fused}$ is then obtained through weighted fusion. In this way, the module can balance spatial details from the original feature and stronger semantic information from the downsampled feature, making it more effective for small traffic signs.

To introduce scale robustness at the feature level, we construct a fused feature pyramid. Specifically, the teacher model processes the original scale image and a 0.5× downsampled image in parallel. After extracting features using the YOLOv11 Backbone, we align and fuse the original scale feature layer $F_{i}$ with the downsampled feature layer $F_{i-1}^{down}$. To achieve adaptive feature fusion, we designed a gated fusion function $g\left(\;\cdot \;\right)$ to dynamically calculate the weights. As shown in Figure 4, for the *i*-th feature layer, we first concatenate the original scale feature $F_{i}$ and the downsampled feature $F_{i-1}^{down}$ along the channel dimension, and we generate the fusion coefficient $\lambda_{i}$ through a lightweight network composed of global average pooling and a Multi-Layer Perceptron (MLP):

(7) $$\lambda_{i}=\sigma \left(MLP\left(AvgPool\left(\left[F_{i},F_{i-1}^{down}\right]\right)\right)\right)$$

where σ is the Sigmoid activation function, used to normalize the weights to the $\left(0,1\right)$. The final fused feature $F_{i}^{fused}$ is obtained by weighted summation:

(8) $$F_{i}^{fused}=\lambda_{i}\;\cdot \;F_{i}+\left(1-\lambda_{i}\right)\;\cdot \;F_{i-1}^{down}$$

This mechanism allows the network to adaptively select whether to retain the detailed information from the original feature layer or the semantic information from the downsampled feature layer, based on the specific scale characteristics of the target.

#### 3.3.2. Candidate Box Selection Based on Confidence Gain

Since the YOLOv11 is a one-stage anchor-free detector, it lacks the candidate box set generated by a Region Proposal Network (RPN). To maintain evaluation under the dense prediction paradigm, we utilize the unique prediction branch structure of YOLO to construct a dual confidence metric.

**Dense Prediction Alignment and Association:** Let *B* be the set of predictions output by the teacher model on the original feature pyramid, and $B_{fused}$ be the set of predictions on the fused feature pyramid. For each candidate box $b_{i}^{\prime }\in B_{fused}$, we first compute its fused confidence and categorize it into positive, ambiguous, or negative groups according to the positive threshold ($\tau_{pos}$) and negative threshold ($\tau_{neg}$). $\tau_{pos}$ is dynamically determined from the mean and standard deviation of fused confidences in the current batch, while $\tau_{neg}$ is fixed at 0.1. For each ambiguous candidate box, we compare it with the corresponding prediction $b_{i}\in B_{reg}$ at the same spatial location.

**Dual Confidence Gain Calculation:** The fused confidence in the original and fused feature pyramids is computed as

(9) $$Conf\left(b_{i}\right)=Cls\left(b_{i}\right)\;\cdot \;IoU\left(b_{i}\right)$$

(10) $$Conf_{\mathrm{fused}\;}\left(b_{i}^{\prime }\right)=Cls_{\mathrm{fused}\;}\left(b_{i}^{\prime }\right)\;\cdot \;IoU_{\mathrm{fused}\;}\left(b_{i}^{\prime }\right)$$

Here, Cls(·) represents the classification confidence predicted by the network. IoU(·) represents the localization confidence predicted by the network.

**Pseudo-Label Generation:** We define the fusion confidence gain as $\Delta Conf=Conf_{fused}\left(b_{i}^{\prime }\right)-Conf_{reg}\left(b_{i}\right)$. If ΔConf exceeds 0.2, the ambiguous proposals are retained, and the proposals $b_{i}^{\prime }$ from the fused feature pyramid are adopted. Therefore, the final candidate box set consists of two parts: positive candidates whose fused confidence exceeds the dynamic positive threshold, and ambiguous candidates which satisfy the fused-confidence gain criterion. In this way, the GFF-PR reduces missed detections of small traffic signs by feature fusion.

### 3.4. Overall Optimization Objective

To fully leverage the proposed semi-supervised framework, we design a comprehensive objective function. The total loss $L_{total}$ for the student model is composed of a supervised loss on labeled data and a multi-task unsupervised loss on unlabeled data:

(11) $$L_{total}=L_{sup}+L_{unsup}=L_{sup}^{cls}+L_{sup}^{loc}+L_{unsup}^{AWFL}+L_{unsup}^{loc}+\mu \;\cdot \;L_{unsup}^{iou}$$

For the labeled data $D_{L}$, we employ standard detection losses. Specifically, $L_{sup}^{cls}$ utilizes the Binary Cross Entropy Loss to minimize the classification error between the prediction and the ground truth class labels. For bounding box regression, $L_{sup}^{loc}$ adopts the Generalized IoU Loss.

For the unlabeled data $D_{U}$, the loss terms are computed using the refined pseudo-labels:

**Adaptive-Weight Focal Loss:** For the unsupervised classification loss, standard semi-supervised methods typically use a binary cross-entropy or standard Focal Loss, and they treat all filtered pseudo-labels as equally important ground truths. This is ineffective because pseudo-labels have different levels of reliability. Moreover, directly using the raw classification confidences as the weight leads to a side-effect: it gives higher weights to head-class pseudo-labels and lower weights to tail class pseudo-labels. As a result, the model pays less attention to the very classes that need more supervision. To address this, we propose the Adaptive-Weight Focal Loss ($L_{\mathrm{unsup}}^{\mathrm{AWFL}}$). Instead of relying on raw classification confidences, we introduce a Class-Adaptive Relative Weight strategy that adjusts the contribution of each pseudo-label based on how much its classification confidence exceeds the class-specific classification-confidence threshold. The formulation is as follows:

(12) $$L_{unsup}^{AWFL}=-\sum_{j}\omega_{j}\;\cdot \;{\left(1-p_{j}\right)}^{\gamma }\log p_{j}$$

where $p_{j}$ is the student model’s classification confidence for the target class, and γ is the focusing parameter. Crucially, $\omega_{j}$ is defined as the ratio between the teacher model’s classification confidence and the class-specific classification-confidence threshold.

(13) $$\omega_{j}=\min\left(\frac{p_{j}^{fused}}{\tau_{c_{j}}^{+}},1.0\right)$$

where $p_{j}^{fused}$ represents the classification confidence of the *j*-th sample in the fused feature pyramid, and $\tau_{c_{j}}^{+}$ denotes the positive classification-confidence threshold corresponding to the predicted class $c_{j}$ of the sample. By defining $\omega_{j}$ as the ratio of the classification confidence to the specific classification-confidence threshold, we ensure that tail class samples are assigned higher importance weights when they exceed their corresponding thresholds. This mitigates the issues caused by the imbalanced class distributions.

**Localization Loss:** Consistent with the supervised branch, we employ GIoU Loss for bounding box regression.

**IoU Consistency Loss:** To improve the student’s estimation of localization quality, we employ Binary Cross Entropy Loss as an auxiliary task.

## 4. Experiments

### 4.1. Datasets

The proposed method is evaluated on the TT100K [42] benchmark dataset released by Tsinghua University and the Tencent Joint Laboratory. TT100K is a large-scale traffic sign dataset collected from real driving environments and covers a wide variety of traffic sign categories under complex road scenes.

Considering the occurrence frequency of traffic signs in urban roads and highway environments, the 84 most common and representative categories are selected. The selected subset exhibits a severe class-imbalance problem. Following the traffic sign distribution in the dataset, categories with more than 500 annotated instances are regarded as head classes, while the remaining categories are treated as tail classes. According to this criterion, the dataset contains 18 head classes and 66 tail classes. The corresponding class distribution is presented in Figure 1.

To enrich the dataset, an additional traffic sign dataset was captured using dashboard cameras and mobile devices across different daytime driving environments (including both urban streets and expressways). The acquisition procedure remains consistent with the TT100K benchmark in terms of imaging conditions and scene characteristics.

To enhance the robustness and generalization capability of the detector, several commonly-used data augmentation techniques were adopted during training, including random image scaling, horizontal flipping, color perturbation, and Mosaic augmentation. After integrating the supplementary data and augmented samples, the complete dataset contains 32,894 images. The dataset is partitioned into training, validation, and testing subsets according to an 8:1:1 ratio. If unspecified, model selection, ablation experiments, and hyperparameter analyses are all performed on the validation set, and the comparison results with the state-of-the-art methods are evaluated on the test set.

### 4.2. Implementation Details

All experiments are implemented in Python 3.7 using the PyTorch 1.13.1 framework. The detailed hardware and software configurations are summarized in Table 1.

The proposed method adopts YOLOv11s as the detector backbone, initialized using the official COCO-pretrained weights. The CLIP ViT-B/32 model pretrained by OpenAI is utilized for zero-shot class distribution estimation.

The input image resolution is resized to 640×640 during both training and inference. Weak augmentation employs multi-scale resizing, while strong augmentation includes Mosaic augmentation, random color jittering, grayscale transformation, Gaussian blur, and three Cutout operations with different parameter settings. Detailed augmentation configurations are summarized in Table 2.

The detector is first pretrained for 300 epochs and then optimized under the semi-supervised setting for 100 epochs. Training is conducted on two NVIDIA Tesla T4 GPUs, with a batch size of 16 for labeled data and 16 for unlabeled data. The Adam optimizer is adopted with an initial learning rate of 0.001. No weight decay is applied during training. The EMA decay parameter α is set to 0.999, and the loss weight coefficient is set to 1. During inference, only the student model is used for evaluation. FPS is measured on a single Tesla T4 GPU under FP32 inference.

### 4.3. Evaluation Metrics

To evaluate the performance of the proposed method, we adopt mAP_50_, $AP_{S}$, $AP_{M}$, and $AP_{L}$ as evaluation metrics.

The mean Average Precision (mAP) is a standard metric for object detection and is computed from the Precision-Recall (P-R) curve. The precision and recall are defined as:

(14) $$mAP=\int_{0}^{1}P\left(R\right)\;dR$$

(15) $$P=\frac{TP}{TP+FP}$$

(16) $$R=\frac{TP}{TP+FN}$$

where $P\left(R\right)$ denotes the precision at a given recall level *R*; TP, FP, and FN denote the numbers of true positives, false positives, and false negatives, respectively. mAP_50_ denotes the mAP values computed at IoU thresholds of 0.5. Also, $AP_{S}$, $AP_{M}$, and $AP_{L}$ represent the average precision for small, medium, and large-scale objects, respectively.

### 4.4. Ablation Study

In the ablation studies, all experiments are conducted under the 10% labeled data setting using Efficient Teacher with the YOLOv11 as the baseline, in order to ensure architectural consistency and fair comparison among different modules. The “Previous Work” in Table 3 denotes the framework inherited from our previous study [27], while the remaining modules correspond to the newly introduced components in the current work.

As shown in Table 3, when introducing the proposed CD-DPLS module on the previous framework, the mAP_50_ further increases from 32.1% to 33.2%, while the mAP_50:95_ improves from 16.9% to 17.5%. This improvement mainly comes from the ability of the CD-DPLS to adjust pseudo-label selection thresholds according to the fused class distribution probabilities.

We further evaluate the effectiveness of the GFF-PR module independently based on the same previous framework. The experimental results show that the GFF-PR improves the mAP_50_ from 32.1% to 33.8%, and the mAP_50:95_ from 16.9% to 17.9%.

To verify the effectiveness of the proposed Adaptive-Weight Focal Loss (AWFL), we replace the original focal loss in Efficient Teacher with AWFL based on the previous framework only. The AWFL improves the performance from 32.1% to 34.9% mAP_50_ and from 16.9% to 19.2% mAP_50:95_.

When the CD-DPLS and GFF-PR are jointly adopted, the proposed method further improves the mAP_50_ from 32.1% to 35.6% and the mAP_50:95_ from 16.9% to 19.8% due to the complementary advantages of dynamic pseudo-label selection and fused-feature refinement. Finally, by combining CD-DPLS, GFF-PR, and AWFL together, the proposed method achieves the best overall performance, reaching 36.3% mAP_50_ and 20.4% mAP_50:95_.

**Figure 5:**
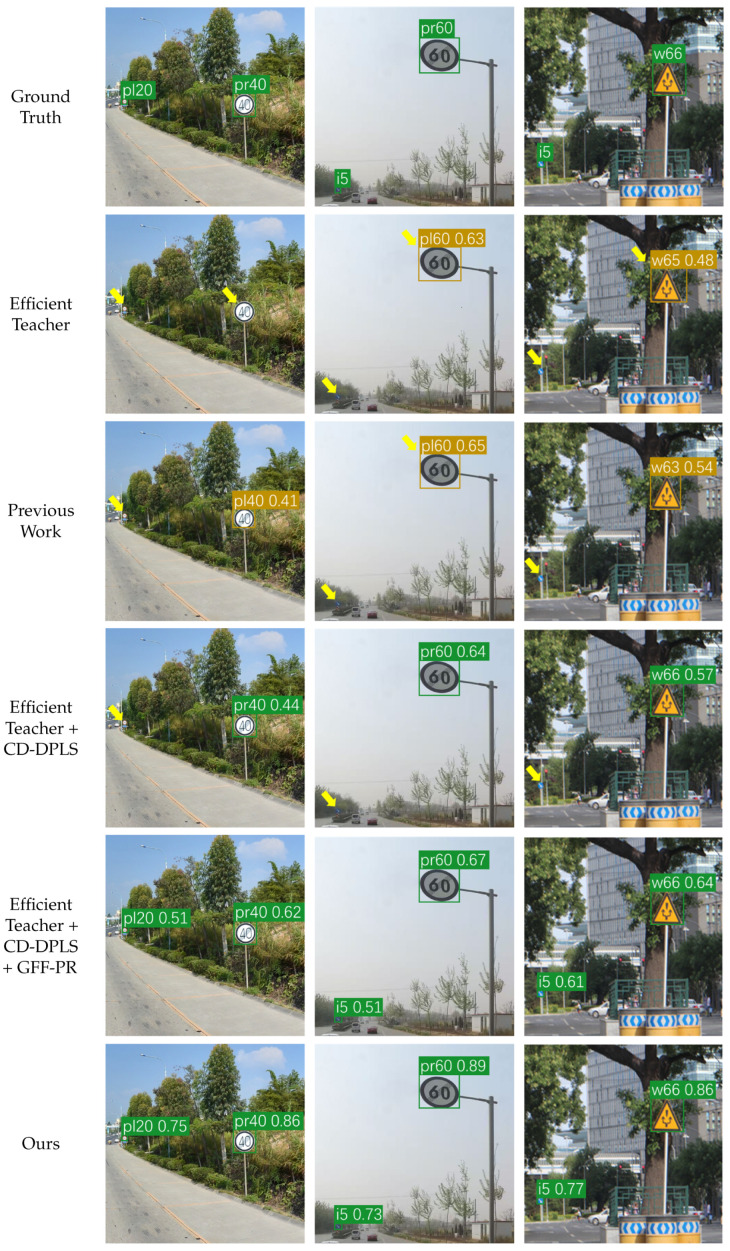
Visual comparison of different components in the proposed method. As the components are gradually introduced, the proposed method shows improvements in tail-class detection, distant small-object detection, as well as prediction confidence.

To visually demonstrate the effectiveness of the proposed components in improving traffic sign detection performance, we conduct an experiment with rare traffic sign classes, as well as small traffic signs. As shown in Figure 5, the baseline model, Efficient Teacher, misses the detection of the tail class “pr40”, while it misclassifies other tail-class objects, such as “pr60” and “w66”. In addition, it also fails to detect small traffic signs in the scenes.

With the CD-DPLS, the model not only detects the previously missed tail-class “pr40”, but it also corrects the misclassification of “pr60” and “w66” by dynamically adjusting the classification-confidence thresholds, thereby improving the detection performance on tail classes. With the GFF-PR, the model can capture small distant targets.

Finally, after adding the AWFL for overall optimization, the model shows higher classification confidence.

**Different Feature Scales:** To verify the impact of fused features in the GFF-PR module, we compare the detection accuracy and inference speed of downsampled feature ($F^{down}$), original feature (*F*), and fused feature ($F^{fused}$). Details are shown in Table 4.

The experimental results show that, although the downsampled feature $F^{down}$ has the fastest inference speed (56 FPS), the resolution reduction of the feature map damages the details of small objects, resulting in only 8.9% Small Object Average Precision ($AP_{S}$), and the lowest overall mAP_50_.

In contrast, the fused feature $F^{fused}$ achieves the best detection performance. Specifically, the $AP_{S}$ increases from 12.4% to 15.4%, demonstrating the effectiveness of the GFF-PR strategy for small traffic sign detection. Although the inference speed decreases to 46 FPS, the model can still satisfy real-time detection requirement. Besides small objects, the fused feature strategy also leads to the detection performance improvements on medium and large objects.

**Pseudo-Label Selection Strategies:** To validate the effectiveness of the proposed CD-DPLS, we compare the model performance under different pseudo-label filtering strategies. Traditional semi-supervised object detection methods typically employ a fixed classification-confidence threshold to filter pseudo-labels; however, this traditional strategy exhibits limitations in the scenario of imbalanced class distributions.

As shown in Table 5, the threshold setting of 0.9 drops the mAP_50_ of tail classes to 15.4%, and it yields only 20.5 valid pseudo-labels per image on average, limiting the overall mAP_50_ to 30.5%. Conversely, the threshold setting of 0.5 leads to the overall mAP_50_ of 33.8%, because of the introduction of incorrect pseudo-labels. However, by dynamically adjusting classification-confidence thresholds based on the fused class distribution probability, the CD-DPLS boosts tail class detection performance to 24.8% and achieves the best overall performance.

To analyze the computational overhead introduced by the CLIP-based class distribution estimation, we compare the training efficiency of different variants.

The experimental results in Table 6 show that incorporating the CLIP prior brings only a slight increase in training cost. Both experiments are trained for 400 epochs. Specifically, the per-epoch training time rises by 0.5 min, and the total training hours increase by 3.2 h. Meanwhile, the mAP_50_ improves from 34.8% to 36.3%. Consequently, the proposed method achieves a favorable trade-off between computational cost and detection accuracy.

**Figure 6:**
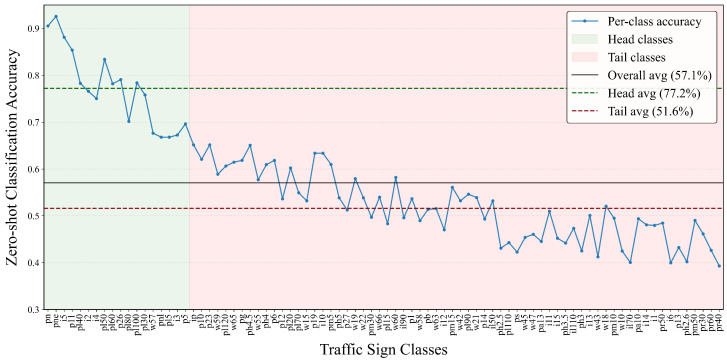
Per-class classification accuracy of CLIP, evaluated across the dataset.

To evaluate CLIP’s reliability, Figure 6 illustrates its per-class zero-shot classification accuracy across the entire dataset. Categories are ordered by descending instance counts to reflect the severe imbalanced class distribution.

As shown in Figure 6, CLIP maintains a stable 77.2% average accuracy on head classes and 51.6% on tail classes. Importantly, our framework uses these predictions solely to estimate a coarse global class distribution prior, not for direct pseudo-labeling. This evaluation confirms that the CLIP-generated prior remains reliable under class imbalance. Consequently, it effectively guides the CD-DPLS module to prevent the detector from overfitting toward majority classes during consistency training, ensuring balanced optimization without introducing severe noise.

To further analyze whether the ignore region contains potentially useful supervision signals, we investigate the confidence distribution of ignored samples and compare the pseudo-label utilization statistics under different threshold strategies.

**Figure 7:**
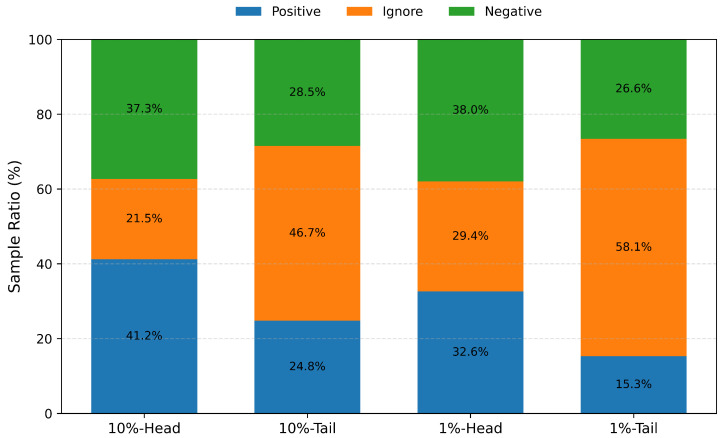
Distribution of positive, ignore, and negative pseudo-label assignments under different labeled-data settings. The ignore ratio is substantially higher for tail classes, especially under the 1% labeled setting, indicating that the teacher model exhibits higher uncertainty on rare categories when labeled supervision is extremely limited.

As shown in Figure 7, the proportion of ignored samples is consistently higher for tail classes than for head classes under both the 1% and 10% labeled settings. This phenomenon becomes more pronounced under the 1% labeled setting, indicating that the teacher model exhibits substantially higher uncertainty for rare categories when labeled supervision is extremely limited.

**Figure 8:**
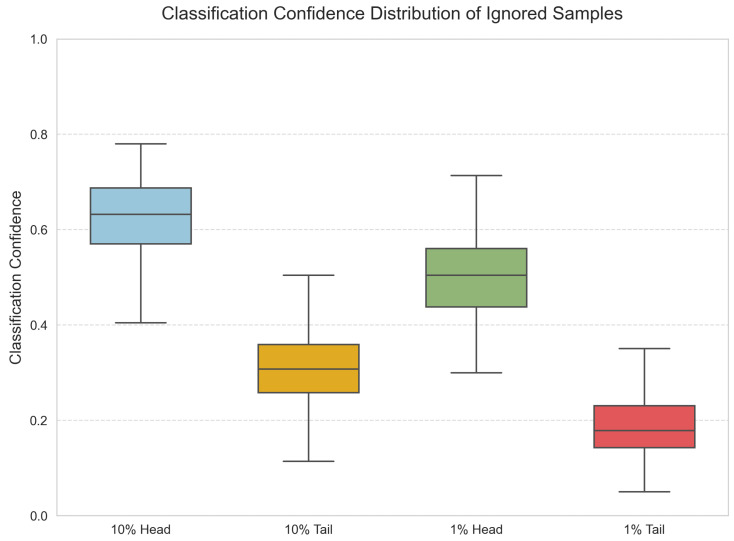
Classification confidence distribution of ignored samples under different labeled-data settings. Tail classes exhibit wider confidence distributions and larger interquartile ranges, indicating the existence of numerous relatively-low-confidence ambiguous samples.

Figure 8 illustrates the confidence distributions of ignored samples. Compared with head classes, ignored samples from tail classes exhibit significantly lower confidence levels across both labeling settings. Consequently, directly adopting a uniform fixed confidence threshold would disproportionately suppress potentially useful supervision signals from tail classes, further exacerbating the class imbalance.

### 4.5. Parameter Sensitivity Analysis

#### 4.5.1. Positive- and Negative-Sample Reliability Ratios

The performance of the CD-DPLS is highly dependent on the threshold settings used for pseudo-label filtering. Specifically, this process is controlled by the positive- and negative-sample reliability ratios $\eta_{1}$ and $\eta_{0}$, which define the proportions of reliable positive pseudo-labels and negative pseudo-labels. To explore the configuration of these two ratios, we conduct a joint grid search within the ranges of $\eta_{1}\in \left[0.80,1.00\right]$ and $\eta_{0}\in \left[0.90,0.99\right]$ under the 10% labeled data setting. The experiment results are detailed in Table 7.

As shown in Table 7, the best detection performance (36.3%) of the proposed method is achieved when $\eta_{1}=0.90$ and $\eta_{0}=0.97$. Here, the optimal settings enable the proposed method to select more reliable pseudo-labels.

As shown in Table 8, the CLIP-based distribution exhibits moderate differences from the labeled-only distribution. The moderate KL and JS divergence together with the high Pearson correlation indicate that the two distributions remain consistent. Although CLIP introduces additional computation, the resulting mAP_50_ improvement from 34.8% to 36.3% suggests that the CLIP can provide beneficial regularization effects, particularly for tail classes.

We also evaluate the reliability of different class distribution estimation strategies, we compare the estimated class distributions with the ground-truth class distribution on the dataset.

As shown in Table 9, the class distribution estimated solely from the labeled data exhibits deviation from the real class distribution due to imbalanced class distribution. The CLIP-based class distribution estimation provides a better approximation of the all class distribution than the labeled-only estimation.

By combining labeled and CLIP-based distributions, the proposed fused distribution achieves the smallest divergence and the highest correlation with the ground-truth distribution. This demonstrates that the CLIP-based prior provides complementary information for dynamic pseudo-label selection.

#### 4.5.2. Class Distribution Fusion Weight

The parameter β serves as a balancing factor between the class distribution probabilities derived from labeled data ($\gamma_{k}^{label}$) and those derived from unlabeled data ($\gamma_{k}^{clip}$). We conduct a sensitivity analysis by varying β from 0.0 to 1.0 with a step size of 0.2 under the 10% labeled data setting.

As shown in Table 10, the detection performance of the proposed method follows an inverted U-shaped trend, indicating that using only labeled or unlabeled class distribution probability is not the best choice. The best detection performance (36.3% mAP_50_) of the proposed method is achieved at β=0.6, demonstrating that combining labeled and unlabeled class distribution probabilities can provide the most accurate fused class distribution probability, which enables better classification-confidence thresholds for each class.

#### 4.5.3. Fusion Confidence Gain Threshold in GFF-PR

The fusion confidence gain threshold ΔConf determines whether ambiguous proposals refined by the GFF-PR module are retained as final pseudo-labels. Specifically, proposals are accepted only when the fused confidence gain exceeds the predefined threshold.

**Figure 9:**
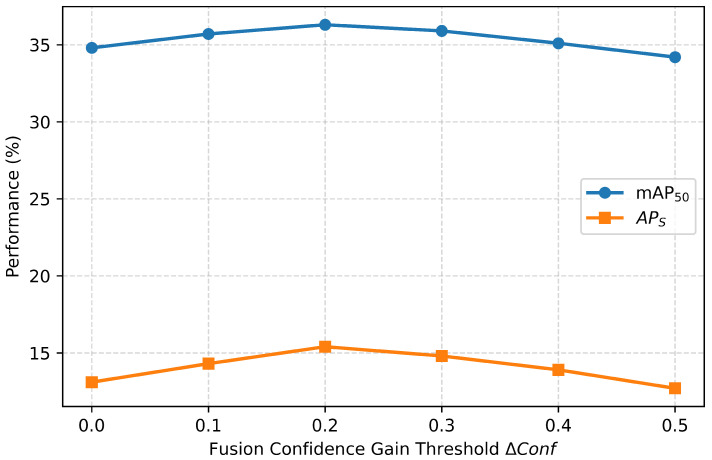
Sensitivity analysis of the fusion confidence gain threshold ΔConf in the GFF-PR module.

To analyze the influence of ΔConf, we conduct a sensitivity study under the 10% labeled setting by varying the threshold from 0.0 to 0.5 with a step size of 0.1. The experimental results are illustrated in Figure 9.

As shown in Figure 9, the detection performance first increases and then decreases as the fusion confidence gain threshold becomes larger. The best detection performance is achieved at ΔConf=0.2, where the proposed method obtains 36.3% mAP_50_ and 15.4% $AP_{S}$. This demonstrates that a moderate fusion confidence gain threshold can effectively balance pseudo-label reliability.

#### 4.5.4. Negative Threshold in GFF-PR

To evaluate the impact of the negative threshold $\tau_{neg}$ in the GFF-PR module, we conduct a sensitivity analysis under the 10% labeled setting, varying $\tau_{neg}$ from 0.05 to 0.50 in increments of 0.05. The experimental results are summarized in Table 11.

As shown in Table 11, the performance peaks at $\tau_{neg}=0.10$, achieving 36.3% mAP_50_, 15.4% $AP_{S}$, and 24.8% mAP_50_(Tail), which balances pseudo-label reliability and sample utilization.

### 4.6. Comparison with the State-of-the-Art Methods

Since this work mainly focuses on improving the detection performance of tail classes and small traffic signs, we additionally report mAP_50_(Tail) and $AP_{S}$ as the key evaluation metrics for long-tail and small-object detection, respectively. To comprehensively evaluate the proposed method, we compare it with representative state-of-the-art semi-supervised object detection methods, including two-stage, one-stage, and Transformer-based detectors. It should be noted that these methods are implemented based on different detector architectures, such as Faster R-CNN, DETR, and YOLO series. Therefore, the comparisons are conducted under the same dataset split, training protocol, hardware environment, and evaluation metrics, rather than strictly identical detector architectures.

To reduce the influence of architectural differences, we further implement the Efficient Teacher framework on both YOLOv5 and YOLOv11 backbones, and we use Efficient Teacher (YOLOv11) as the primary one-stage baseline. Compared with Efficient Teacher (YOLOv11), our method improves the mAP_50_ from 26.7% to 34.9% under the 10% labeled setting, demonstrating that the performance gain mainly originates from the proposed modules rather than solely from the backbone upgrade.

As shown in Table 12, our method achieves the best overall performance across all labeled data ratios. Under the 10% labeled setting, our method achieves 34.9% mAP_50_.

Furthermore, under the 10% labeled setting, the proposed method achieves the best performance on both tail-class detection and small-object detection, reaching 23.7% mAP_50_(Tail) and 14.6% $AP_{S}$, respectively. These results demonstrate that the proposed CD-DPLS and AWFL effectively alleviate the imbalanced class distributions, while the GFF-PR enhances the detection performance of small traffic signs.

Regarding inference efficiency, our method achieves 42.1 FPS, which is faster than the two-stage and end-to-end methods while maintaining superior detection accuracy. Although the proposed modules introduce additional computational overhead compared with the original Efficient Teacher framework, the substantial performance improvement demonstrates a trade-off between accuracy and efficiency.

Table 13 compares our proposed method with state-of-the-art semi-supervised object detection methods under 10% labeled semi-supervised setting and the 100% labeled fully-supervised setting of the traffic sign training set. Under both the 10% labeled semi-supervised setting and the 100% labeled fully-supervised setting, our method achieves the best detection performance. Meanwhile our method’s highest mAP_50_(Tail) and $AP_{S}$ further verify its effectiveness for long-tail and small-object traffic sign detection. It shows that the improvements on tail classes and small objects do not degrade the overall detection performance.

### 4.7. Visualization

**Figure 10:**
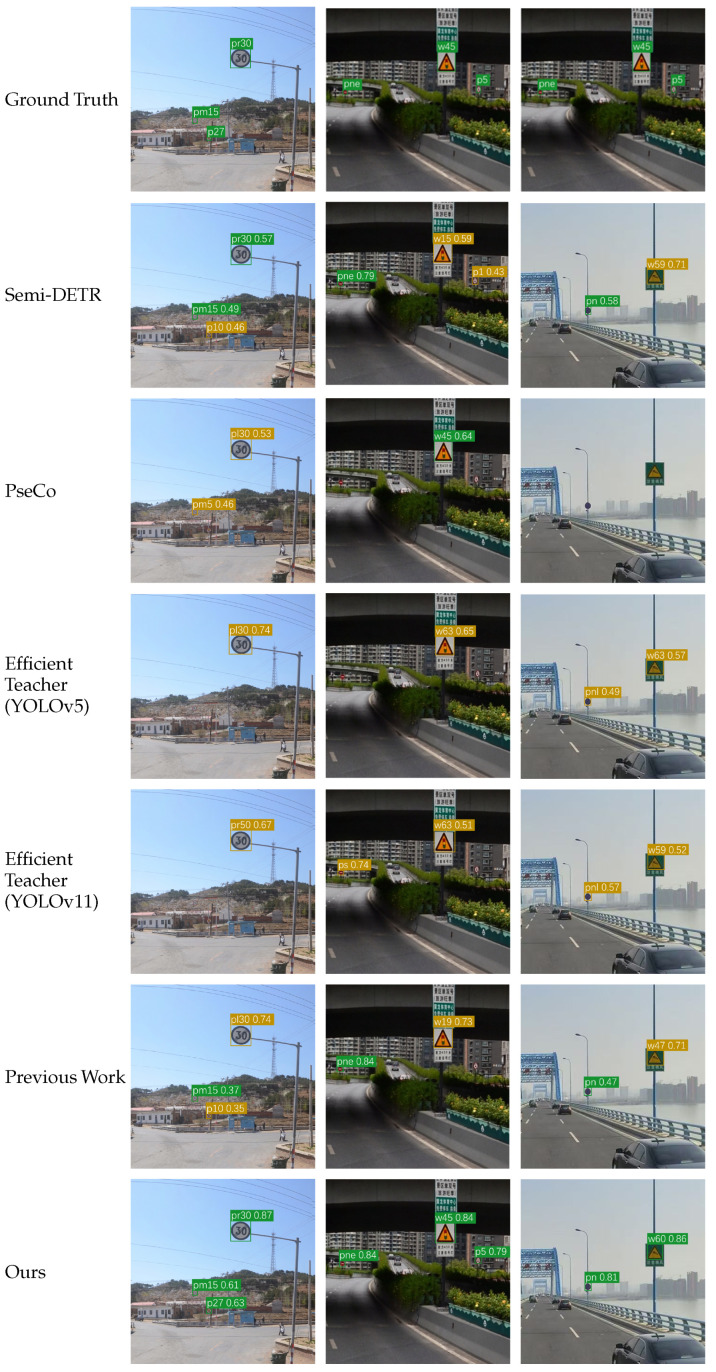
Qualitative comparison between the proposed method and the state-of-the-art semi-supervised detection methods. Green boxes indicate the correct predictions, and yellow boxes indicate the wrong predictions.

To demonstrate the effectiveness of the proposed semi-supervised detection method, we select several representative cases of small objects and tail classes for qualitative comparison. Figure 10 presents the detection results of our method, the ground truth, our previous work, and several representative semi-supervised methods. These methods include Semi-DETR and the PseCo (representing the best detection performances in end-to-end and two-stage categories, respectively), as well as our baseline, Efficient Teacher, implemented on both YOLOv5 and YOLOv11. In addition, we also include our previous work [27] for comparison to further demonstrate the effectiveness of the newly introduced components in the current framework.

**(1) Small-object detection:** As shown in the distant traffic sign cases, small targets have only a few pixels in the image, so they are easily ignored in deep networks. Specifically, Efficient Teacher and the PseCo exhibit missed small-object detections. Our previous work can improve the detection performance of several small targets; however, it still suffers from false detection and missed detection. In addition, all the comparison methods except ours also suffer from false detections. In contrast, with the help of the GFF-PR, our method can better recover small objects by using fused features.

**(2) Tail-class detection:** As shown in Figure 10, all the comparison methods except ours misclassify the tail class objects as other classes. Furthermore, the PseCo also exhibit missed detections for tail-class objects. Although our previous work improves the feature representation ability, it still suffers from incorrect predictions on several tail-class traffic signs due to the class-imbalance problem. In contrast, the CD-DPLS retains more pseudo-labels for tail classes by dynamically adjusting the classification-confidence thresholds according to the fused class distribution probability, while the AWFL mitigates the issues caused by the imbalanced class distribution. As shown in the last row of Figure 10, our proposed method predicts the correct classes with higher confidence for tail-class objects such as “pr30”, “w45”, and “w60”.

## 5. Conclusions

In this paper, we propose a semi-supervised traffic sign detection method to address two challenges in traffic scenes: imbalanced class distributions and missed small-object detections. The proposed CD-DPLS combines class distribution probabilities from labeled and unlabeled data to dynamically adjust pseudo-label selection, increasing the detection performance on tail classes. In addition, the GFF-PR improves the detection of small objects by leveraging fused features to re-evaluate ambiguous proposals. Finally, we introduce the AWFL, which assigns adaptive weights to traffic sign classes, to mitigate the issues caused by the imbalanced class distributions.

Experiments show that the proposed method outperforms the existing state-of-the-art semi-supervised detection methods. It also maintains a real-time inference speed of 45.8 FPS, demonstrating a balance between detection accuracy and efficiency. The experimental results suggest that the proposed method is an effective solution for real-world intelligent transportation systems.

Although the proposed AWFL adaptively adjusts pseudo-label weights according to the confidence-threshold ratio, the current framework still utilizes a heuristic proxy for pseudo-label reliability rather than explicit uncertainty estimation. In extremely low-label and long-tail scenarios, pseudo-labels with confidence values slightly above the selection threshold may still contain substantial uncertainty. Therefore, future work will explore incorporating uncertainty-aware semi-supervised learning strategies, such as predictive variance estimation and uncertainty-guided pseudo-label selection, to further improve pseudo-label reliability and training stability.

## Figures and Tables

**Table 1:** Hardware and software configurations in the experimental setup.

Component	Specification
Operating System	Ubuntu 16.04
CPU	Intel(R) Xeon(R) Gold 5218 CPU @2.30 GHz
Memory	6 4GB
GPU	Tesla T4 × 2
VRAM	15 GB × 2
Python	3.7
PyTorch	1.13.1

**Table 2:** Data augmentation configurations used in semi-supervised training.

Augmentation	Probability	Configuration
Multi-scale resizing	1.0	Scaling range [0.1, 1.9]
Mosaic augmentation	1.0	Default YOLO Mosaic strategy
Color jittering	1.0	Brightness [0.6, 1.4], saturation [0.3, 1.7], hue [−0.015, 0.015]
Grayscale transformation	0.2	–
Gaussian blur	0.5	Gaussian kernel: $\sigma_{x}=0.1$, $\sigma_{y}=2.0$
Cutout1	0.7	Scale range [0.05, 0.2], aspect ratio [0.3, 3.3]
Cutout2	0.5	Scale range [0.02, 0.2], aspect ratio [0.1, 6]
Cutout3	0.3	Scale range [0.02, 0.2], aspect ratio [0.05, 8]

**Table 3:** Ablation study of different components under the 10% labeled data setting. “Previous Work” denotes our previous method, “CD-DPLS” denotes the Class Distribution-based Dynamic Pseudo-Label Selection module, “GFF-PR” denotes the Gated Feature Fusion-based Proposal Refinement strategy, and “AWFL” denotes the Adaptive-Weight Focal Loss.

Previous Work [27]	CD-DPLS	GFF-PR	AWFL	mAP_50_	mAP_50:95_
				27.8%	14.8%
✓				32.1%	16.9%
✓	✓			33.2%	17.5%
✓		✓		33.8%	17.9%
✓			✓	34.9%	19.2%
✓	✓	✓		35.6%	19.8%
✓	✓	✓	✓	36.3%	20.4%

**Table 4:** Performance comparison of different feature scale. We compare the detection accuracy and inference speed utilizing strictly downsampled features ($F^{down}$), original scale features (*F*), and the proposed fused features ($F^{fused}$).

Feature Level	mAP_50_	${\mathrm{AP}}_{S}$	${\mathrm{AP}}_{M}$	${\mathrm{AP}}_{L}$	FPS
$F^{down}$	32.8%	8.9%	22.1%	36.1%	56
*F*	34.1%	12.4%	24.5%	33.5%	52
$F^{fused}$	36.3%	15.4%	25.8%	36.5%	46

**Table 5:** Performance comparison of different pseudo-label selection strategies. We compare the detection accuracy on head and tail classes and the dynamic thresholding process from initial candidates to final valid pseudo-labels utilizing fixed thresholds (0.9, 0.5) and the proposed CD-DPLS. “All” means all the classes in the dataset, “Head” means the head classes, and “Tail” means the tail classes.

Threshold Strategy	mAP_50_(All)	mAP_50_(Head)	mAP_50_(Tail)	Avg. Initial Candidates	Avg. Pseudo-Labels
0.9 (Fixed)	30.5%	58.2%	15.4%	138.6	20.5
0.5 (Fixed)	33.8%	52.1%	20.6%	162.4	45.8
CD-DPLS (Ours)	36.3%	60.5%	24.8%	145.2	32.4

**Table 6:** Computational overhead analysis of CLIP-based class distribution estimation.

Method	Training Time/Epoch	Total Training Time	mAP_50_
Ours w/o CLIP Prior	12.4 min	82.6 h	34.8%
Ours w/ CLIP Prior	12.9 min	85.8 h	36.3%

**Table 7:** Sensitivity analysis of positive- and negative-sample reliability ratios. The table reports the mAP_50_ performance under varying combinations of $\eta_{1}$ and $\eta_{0}$.

$\eta_{1}\setminus \eta_{0}$	0.90	0.93	0.95	0.97	0.99
0.80	33.8%	34.2%	34.5%	34.2%	33.9%
0.85	34.2%	34.6%	34.9%	34.9%	34.5%
0.90	34.5%	34.9%	35.1%	36.3%	35.3%
0.95	34.3%	34.7%	34.8%	35.2%	34.9%
1.00	33.9%	34.3%	34.5%	34.7%	34.4%

**Table 8:** Discrepancy between the CLIP-based and labeled-only class distributions. Lower KL and JS divergence indicate higher similarity between the two distributions, while a higher Pearson correlation coefficient indicates stronger linear consistency.

Comparison	KL Divergence	JS Divergence	Pearson Correlation
CLIP vs. Labeled	0.241	0.132	0.854

**Table 9:** Quality comparison of different class distribution estimation strategies. Lower KL and JS divergences indicate distributions closer to the ground-truth distribution, while a higher Pearson correlation coefficient indicates stronger consistency with the real class distribution.

Distribution Source	KL Divergence	JS Divergence	Pearson Correlation
Labeled-only Distribution	0.214	0.118	0.781
CLIP-only Distribution	0.173	0.096	0.842
Fused Distribution (β=0.6)	0.109	0.061	0.917

**Table 10:** Sensitivity analysis of the class distribution fusion weight β in the CD-DPLS. β=0 represents using only class distribution probabilities derived from unlabeled data, while β=1 represents using only the class distribution probabilities derived from labeled data.

β Value	0.0	0.2	0.4	0.6	0.8	1.0
mAP_50_	33.9%	35.6%	36.1%	36.3%	35.8%	35.1%

**Table 11:** Sensitivity analysis of the negative threshold $\tau_{neg}$ in the GFF-PR module under the 10% labeled setting.

$\tau_{\mathrm{neg}}$	mAP_50_	${\mathrm{AP}}_{S}$	mAP_50_(Tail)
0.05	35.9%	15.1%	24.3%
0.10	36.3%	15.4%	24.8%
0.15	36.1%	15.2%	24.5%
0.20	35.7%	14.9%	24.0%
0.25	35.4%	14.6%	23.7%
0.30	35.0%	14.3%	23.2%
0.35	34.6%	14.0%	22.7%
0.40	34.1%	13.6%	22.1%
0.45	34.0%	13.4%	21.8%
0.50	33.9%	13.1%	21.4%

**Table 12:** Comparison with state-of-the-art semi-supervised object detection methods on the traffic sign dataset. All methods are evaluated under the same dataset split, training schedule, and evaluation metrics. FPS, mAP_50_(Tail), and $AP_{S}$ are reported under the 10% labeled setting.

Category	Method	Backbone	mAP_50_				mAP_50_(Tail)	${\mathrm{AP}}_{S}$	FPS
			1%	2%	5%	10%			
End-to-end	Omni-DETR [43]	ResNet-50	8.7%	14.4%	19.2%	27.4%	17.3%	9.5%	10.7
	Semi-DETR [32]	ResNet-50	9.2%	15.5%	20.6%	28.1%	18.0%	10.1%	9.1
Two-stage	STAC [28]	ResNet-50	5.1%	6.9%	12.1%	20.4%	12.1%	6.1%	14.4
	Unbiased Teacher [29]	ResNet-50	6.4%	11.8%	15.9%	23.6%	15.4%	7.9%	16.8
	PseCo [44]	ResNet-50	7.7%	14.0%	18.2%	26.5%	17.5%	10.2%	15.7
	Humble Teacher [30]	ResNet-50	6.1%	8.8%	14.4%	23.8%	14.5%	7.4%	17.8
	SimLTD [16]	ResNet-50	7.6%	12.6%	17.9%	25.0%	20.8%	8.7%	17.3
One-stage	Efficient Teacher [45]	YOLOv5	6.9%	11.8%	14.9%	22.3%	14.7%	8.3%	50.5
	Efficient Teacher (baseline)	YOLOv11	8.2%	13.9%	18.5%	26.7%	19.7%	11.6%	49.3
	Unbiased Teacher v2 [46]	ResNet-50	6.9%	9.3%	15.4%	22.6%	15.1%	8.3%	48.8
	One Teacher [47]	YOLOv11	7.8%	13.2%	18.3%	25.8%	18.3%	9.8%	44.8
	Dense Teacher [48]	YOLOv11	8.2%	13.5%	18.6%	26.2%	18.7%	10.6%	46.1
	Previous Work [27]	YOLOv11	9.9%	16.9%	22.4%	30.7%	21.5%	12.9%	45.8
	Ours	YOLOv11	10.8%	18.1%	25.2%	34.9%	23.7%	14.6%	42.1

**Table 13:** Comparison with state-of-the-art semi-supervised object detection methods under 10% labeled semi-supervised setting and the 100% labeled fully-supervised setting of the traffic sign training set. The 100% labeled fully-supervised setting uses the whole training set (80% of the whole dataset) with all annotations available.

Method	10% Labeled Semi-Supervised				100% Labeled Fully-Supervised			
	mAP_50_	mAP_50:95_	mAP_50_(Tail)	*AP_S_*	mAP_50_	mAP_50:95_	mAP_50_(Tail)	*AP_S_*
Omni-DETR [43]	27.4%	14.8%	17.3%	9.5%	44.6%	25.1%	29.4%	18.7%
Semi-DETR [32]	28.1%	15.4%	18.0%	10.1%	45.8%	25.8%	30.3%	19.5%
STAC [28]	20.4%	10.7%	12.1%	6.1%	40.7%	22.3%	24.8%	15.3%
Unbiased Teacher [29]	23.6%	12.6%	15.4%	7.9%	42.5%	23.6%	27.1%	16.9%
PseCo [44]	26.5%	14.3%	17.5%	10.2%	44.2%	24.9%	29.7%	19.1%
Humble Teacher [30]	23.8%	12.5%	14.5%	7.4%	42.1%	23.2%	26.4%	16.4%
SimLTD [16]	25.0%	13.5%	20.8%	8.7%	43.8%	24.4%	31.5%	17.5%
Efficient Teacher [45]	22.3%	12.0%	14.7%	8.3%	43.4%	24.1%	27.5%	17.2%
Efficient Teacher (baseline)	26.7%	14.6%	19.7%	11.6%	46.8%	26.5%	32.8%	21.8%
Unbiased Teacher v2 [46]	22.6%	12.2%	15.1%	8.3%	43.0%	23.9%	27.2%	17.1%
One Teacher [47]	25.8%	13.9%	18.3%	9.8%	45.6%	25.7%	31.2%	20.0%
Dense Teacher [48]	26.2%	14.2%	18.7%	10.6%	46.1%	26.1%	31.9%	20.8%
Previous Work [27]	30.7%	16.8%	21.5%	12.9%	47.3%	26.8%	33.4%	22.5%
Ours	34.9%	19.4%	23.7%	14.6%	49.6%	28.7%	35.6%	24.3%

## Data Availability

The data presented in this study are available upon request from the corresponding author. The data are not publicly available due to privacy restrictions.
